# The Long-Standing Problem of Proliferative Retinopathies: Current Understanding and Critical Cues

**DOI:** 10.3390/cells14141107

**Published:** 2025-07-18

**Authors:** Maurizio Cammalleri, Paola Bagnoli

**Affiliations:** Department of Biology, University of Pisa, 56123 Pisa, Italy; paola.bagnoli@unipi.it

**Keywords:** retinal neovascular diseases, anti-VEGF treatment, alternative strategy, neuroprotection, updated methodologies and assessment, translational medicine

## Abstract

Retinal ischemia is implicated in ocular diseases involving aberrant neovessel proliferation that characterizes proliferative retinopathies. Their therapy still remains confined to the intravitreal administration of anti-vascular endothelial growth factor (VEGF) medication, which is limited by side effects and progressive reduction in efficacy. Mimicking neovascular diseases in rodents, although of great help for translating fundamental mechanistic findings and assessing therapeutic potential in humans, is limited by the rodent’s short life span, which prevents retinal vessel proliferation over time. However, the oxygen-induced retinopathy (OIR) model, which mimics retinopathy of prematurity, seems to meet some criteria that are common to proliferative retinopathies. The present review provides insight into preclinical models and their suitability to mimic proliferative retinopathies. Further considerations will be applied to emerging approaches and advanced methodologies for the management of proliferative retinopathies, leading to the identification of new therapeutic targets, including our contribution in the field. Major emphasis is given to the possibility of using systemic therapies either alone or in combination with intravitreal anti-VEGF administration to maximize clinical benefits by combining drugs with different modes of action. This review is concluded by an in-depth discussion on future advancements and a critical view of preclinical finding translatability. Despite the major effort of preclinical and clinical research to develop novel therapies, the blockade of VEGF activity still remains the only treatment for proliferative retinopathies for more than twenty years since its first therapeutic application.

## 1. Introduction

Physiological angiogenesis leads to the formation of a hierarchical vessel network that is designed by intrinsic and extrinsic signals to establish the specific vascular phenotype necessary to meet the needs of the local tissue. Pathological angiogenesis, in contrast, does not replicate physiological angiogenesis but results in diseased perfusion due to abnormal vessel growth and structural abnormalities, including vessel leakage and a tendency to bleed.

Pathological angiogenesis is a major cause of human blinding diseases, and extensive effort has been invested in the study of mechanisms underlying the neovascular diseases of the retina to better address their potential treatments. Over the past three decades, significant progress has been made in the treatment of eye diseases and preventable blindness, but 1.1 billion people still live with vision impairment.

With the aging of the population, vision loss has become one of the most serious public health concerns, mainly considering that the worldwide population aged 60 years and older will double (2.1 billion) by 2050 [1]. Although defects of the anterior eye mainly contribute to vision loss prevalence [2], age-related retinal diseases, including wet age-related macular degeneration (neovascular AMD, nAMD) and proliferative diabetic retinopathy (PDR), account for nearly 12% of all cases of visual impairment worldwide. New vessel proliferation is a common event in nAMD and PDR, with abnormal blood vessels growing in the sub-retinal pigment epithelium (RPE) cell spaces, thereby forming macular neovascularization in nAMD. In PDR, insufficient blood flow promotes new vessel growth that can extend into the vitreous cavity, thus clouding the vitreous and compromising vision. Uncontrolled retinal angiogenesis that characterizes both nAMD and PDR is similar to what occurs in retinopathy of prematurity (ROP), a serious morbidity affecting preterm infants. Despite its low incidence, ROP remains a major cause of childhood blindness due to the increasing number and survival rate of extremely preterm infants.

Proliferative retinopathies, whether age-related, such as PDR and nAMD, or premature, such as ROP, are characterized by the same stages of progression, although they depend on different causes intimately associated with decreased levels of tissue oxygenation. PDR, in fact, is induced by hyperglycemia that leads to vascular damage, resulting in an ischemic environment, while AMD is initiated by low oxygen tension due to reduced blood flow to the outer retina. Finally, ROP is induced by hypoxia due to the insufficient vascularization of the developing retina following preterm exposure to high oxygen tension. In ROP, in particular, early vaso-obliteration in the peripheral retina leads to hypoxia, which causes the excessive and pathological growth of the superficial vessels towards the vitreous cavity. The consequent detachment of the retina occurs secondary to the contraction of fibrous tissue, which often accompanies neovascularization [3]. In the case of AMD, aging is perhaps the most significant factor, but additional risk factors, including systemic diseases and unhealthy habits, may accelerate the aging process of the retina. Among AMD patients, almost 10% develop choroidal neovascularization (CNV), which is the hallmark of neovascular AMD (nAMD) [4]. CNV involves neovessel growth from the choroid to the subretinal space through a break in Bruch’s membrane, a barrier between the retina and the choroid.

Specialized disease models and advanced methodologies may improve our understanding of disease pathology, thus helping in the identification of new therapeutic targets. In this respect, the rodent retina has been used extensively to analyze both physiological and pathological angiogenesis, mostly because its vasculature develops postnatally, thus allowing for experimental manipulation. In the present review, our goal is to provide insight into preclinical models and their suitability to mimic proliferative retinopathies. For instance, the models of intravitreal injections of vascular endothelial growth factor (VEGF) medication can be used to mimic ischemic retinopathies, although hemorrhages and edema can complicate tissue modeling [5]. In addition, genetic mouse models of diabetes and its complications, including Akimba and Akita, are used as models of spontaneous hyperglycemia, therefore exhibiting features relevant to DR, although mimicking early retinal complications [6]. Furthermore, additional non-rodent models have been used to replicate proliferative retinopathies, although no single animal model fully encompasses the entire DR pathogenesis of the human eye, including PDR. In the present review, special attention will be given to the mouse model of oxygen-induced retinopathy (OIR), which comes as close as possible to a model of neovessel proliferation in the retina. Further considerations will be applied to emerging approaches and advanced methodologies for the evaluation and management of proliferative retinopathies. The major limitations of models and technical approaches will also be discussed.

## 2. In Vivo Models for Mimicking Neovascular Diseases of the Retina

Experimental studies are those where researchers introduce an intervention and study its effects. Interventions aimed at mimicking human diseases are the foundation for elucidating disease mechanisms, identifying therapeutic targets, and developing treatment strategies. Rodent models have been extensively employed for disease modeling as their characteristics may allow accurate and effective investigations to address the complexity of human diseases.

Pathological angiogenesis in the retina is recognized as one of the main leading causes of blindness, and much effort is aimed at developing animal models that mimic proliferative retinopathies. Animal models, despite remaining the best fit in preclinical studies, are limited by their interspecies differences and poor prediction of human physiology and pathology. In this respect, the high failure rate of drug development using animal models of human diseases requires a marked shift towards bioengineered models, including organoids, tissue models, and organs-on-chips [7].

Although advancements in alternative methodologies will transform biomedical research, animal models still provide a comprehensive perspective of the entire organism that alternative models cannot yet fully replicate.

### Proliferative Retinopathies

In vasoproliferative diseases of the eye, distinct vascular beds are affected, with an impaired vascular network in the choroid that characterizes nAMD and retinal vessel proliferation that identifies proliferative retinopathies, including PDR and ROP. In nAMD, choroidal vessels between the retina and the sclera abnormally grow under the macula. They leak blood and fluid that collect between the RPE and the retina, thus affecting the structure of the macula and ultimately leading to vision loss or distortion [8]. PDR is instead characterized by the growth of new vessels on the surface of the retina towards the vitreous cavity. These new vessels are fragile and prone to bleeding and leaking, thus resulting in retinal edema. As in nAMD, in PDR, retinal vessel proliferation takes years to develop and cannot be mimicked by mouse models of diabetes. In fact, the short lifespan of rodents limits the duration of DR, therefore replicating its early stages but failing to mimic the advanced PDR, which involves neovessel proliferation in the retina. Indeed, rodent models of diabetes, either streptozotocin-induced type 1 diabetic or genetically modified type 2 diabetic, reproduce most aspects of the early stages of DR without developing retinal vessel proliferation [9]. On the other hand, OIR mice, although characterized by retinal neovascularization, differ from the clinical scenario because they lack the metabolic complications of diabetes.

**Figure 1:**
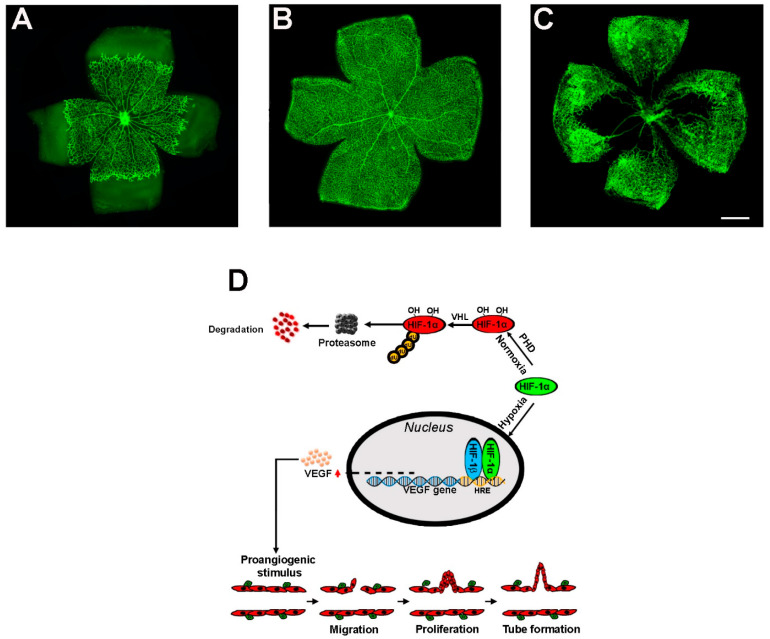
Angiogenic processes in the retina. (**A**) During maturation, a decreasing oxygen gradient from the center to the periphery leads to progressive vessel growth in response to VEGF release. (**B**) Around postnatal day (PD) 8, the superficial vessels cover the entire retina under normoxic conditions. (**C**) In the OIR model, the response to hypoxia induced by vaso-obliteration in the central retina is characterized by excessive and pathological vascular growth that occurs in the mid-periphery (scale bar: 1 mm). Images refer to retinas stained with fluorescein isothiocyanate (FITC)-conjugated isolectin B4 that are part of a database collecting previously published results. (**D**) At the molecular level, hypoxia suppresses the activity of PHD, an enzyme that hydroxylates specific proline residues in an HIF-1α domain, which is sensitive to oxygen levels. Thus, preventing HIF-1α hydroxylation does not result in the VHL-induced proteasomal degradation of HIF-1α, leading to its dimerization with HIF-1β, which generates the active form of HIF-1. In the following cascade, HIF-1 binds to HRE, a DNA sequence that promotes the transcription of several target *genes*. Of these, the VEGF gene leads to the production of VEGF proteins that bind to their receptors, thus triggering the angiogenic cascade, which culminates in neovessel proliferation. HIF-1, Hypoxia-inducible factor-1; HRE, hypoxia response element; PHD, prolyl hydroxylase domain protein; VEGF, vascular endothelial growth factor; VHL, Von Hippel–Lindau. Modified from [10].

Neovascular response to low oxygen tension is central to maintaining oxygen homeostasis and is triggered by a complex network of interconnected molecular pathways involving hypoxia-inducible factors (HIFs) that regulate the expression of *genes* associated with several physiopathological processes, including angiogenesis. As represented in Figure 1, the superficial vessels of the retina undergo drastic changes in response to hypoxia through basic mechanisms, including HIF-1 and VEGF.

## 3. Mouse Modeling of Proliferative Retinopathies

The development of reliable and human-relevant animal models of angiogenesis-related ocular diseases is a valuable research tool. The mouse is the most commonly used mammalian model for a number of reasons, including its small body size, genetic resources, relatively short life cycle, frequent mating, reasonable cost, and ease of maintenance in captivity. The small weight of mice allows for a reduction in the amount of compounds needed for the experiment, although the dose translation from mice to humans is complicated by conversion parameters for appropriate dosage [11]. The widespread availability of transgenic, mutant, or knockout strains makes mice a very attractive model for studying retinal diseases and targeted therapeutic treatments. Despite ever-growing investigations, the mouse visual system, however, remains a limited resource due to its poor capacity in visual processing compared to additional sensory skills, including odor information. On the other hand, the mouse retina has been extensively used to investigate physiological and pathological angiogenesis, mostly because of the postnatal development of the retinal vasculature that allows for the study of both the formation and regression of blood vessels [12]. In fact, many key early events of retinal development, including retinal vessel maturation that occurs during gestation in humans, are accessible for investigation in postnatal rodents. On the other hand, rodent models are indeed suitable for replicating the early, non-proliferative stages of retinopathy, but their short life span prevents them from mimicking the vascular complications that are associated with the advanced, proliferative stages, which occur in humans. In addition, the lack of a cone-rich macula for high-acuity vision prevents the unveiling of the mechanisms underlying macular degeneration, thus preventing the uncovering of the full spectrum of nAMD [13]. Blood supply to the macula derives from the choriocapillaris, a dense capillary network at the posterior pole of the eye, which provides nutrients to photoreceptors and the RPE, from which it removes waste material. Therefore, intervening in choroidal circulation in mouse models may help reproduce the altered choroidal blood flow that characterizes AMD patients, supporting the translatable nature of the CNV model. In this model, the break of Bruch’s membrane by laser photocoagulation compromises retinal homeostasis, thus promoting the production of angiogenic factors and resulting in choroidal vessel proliferation similar to what is seen in nAMD. The laser-induced CNV model, though useful, has some limitations, as the choroid-retinal environment in the mouse does not mimic the pathologic changes in human AMD. In addition, laser-induced angiogenesis does not involve the same pathways as those characteristic of late-occurring human AMD [14]. Moreover, nAMD exhibits a multifactorial etiology involving oxidative stress, hypoxia, inflammation, and angiogenesis driven by a combination of genetic and environmental risk factors [13]. In addition to AMD, DR is a complex and progressive disease characterized by a range of pathological alterations, starting with microangiopathy that leads to increased vascular permeability at the level of the blood–retinal barrier (BRB), which then causes diabetic macular edema associated with microaneurysms, followed by pathological angiogenesis leading to vitreous hemorrhage and tractional retinal detachment and finally culminating in visual loss [15]. The models of pharmacologic DR are classically performed by the administration of the antibiotic streptozotocin, which is known to damage pancreatic cells and cause hyperglycemia, thus mimicking type 1 diabetes. The pathogenesis of DR is driven by high blood sugar that ultimately leads to vascular damage due to several mechanisms, of which the increased production of reactive oxygen species leads to oxidative stress that drastically impairs retinal vessel function. As a consequence, retinal ischemia promotes anomalous neovascularization in the posterior segment of the eye. Alternatively, rodent genetic models of DR, although replicating DR complexity, are not free from limitations [16]. Indeed, vascular and neuronal dysfunction, both triggered by genetic and environmental components, strongly support the difficulty that a single animal model may replicate the entire DR pathogenesis.

### The OIR Model

**Figure 2:**
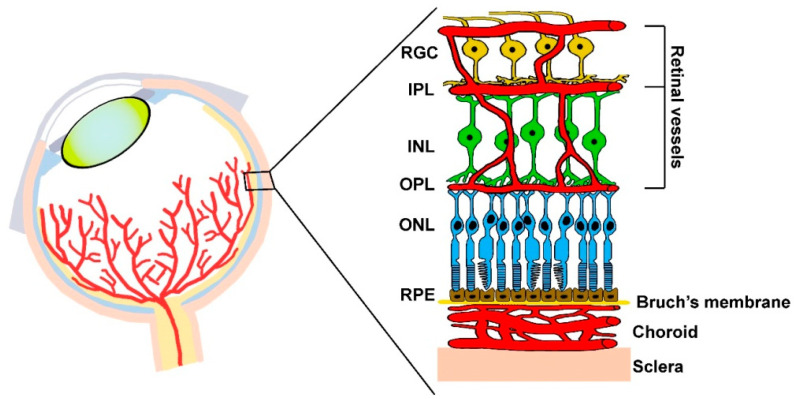
Vessel plexus among retinal layers with respect to distinct retinal cells. The primary plexus at the retinal surface meets the metabolic demands of RGCs that first appear during embryogenesis. Deeper plexus development is initiated from the superficial plexus in concomitance with the progressive maturation of retinal cells in the inner and outer retinal layers. Lastly, formed photoreceptor cells are supplied by the choroidal vasculature, which provides oxygen and nutrients to the outer retina from which it is separated by Bruch’s membrane. RGC, Retinal ganglion cell; IPL, inner plexiform layer; INL, inner nuclear layer; OPL, outer plexiform layer; ONL, outer nuclear layer; RPE, retinal pigment epithelium. Adapted from [19].

The mouse model of OIR is most commonly used to replicate ROP and has provided a critical understanding of its underlying mechanisms, including dysregulated signaling pathways leading to pathologic angiogenesis [17]. In the OIR model, delayed maturation of the retinal vasculature represents a great advantage, as vessel response to changing oxygen levels occurs postnatally and can be easily detected and quantified. The postnatal maturation of retinal vasculature goes hand-in-hand with the postnatal development of retinal circuitry that is designed by activity-independent gene expression and vision-dependent remodeling. In fact, the superficial vascular plexus forms during the first postnatal week in concomitance with retinal ganglion cell (RGC) integration into the retinal circuitry, while the three additional vascular networks become established up to the third postnatal week, depending on the coordinated signaling between developing retinal cell types, their metabolic requirements, and the external supply of oxygen and nutrients [18]. In Figure 2, the three vascular plexuses are represented with respect to retinal layers with the superficial plexus, which is established in concomitance with RGC maturation.

The inability to reproduce retinal vessel proliferation may be overcome by the use of mouse models of OIR that reproduce the events occurring during ROP, but these models can be extended to mimic vessel proliferation, which characterizes additional proliferative retinopathies. In the OIR model, as established in the early nineties by Smith et al. [20], the formation of pathological neovascularization can be induced by an imbalance between pro- and anti-angiogenic factors. In particular, alternating hyperoxia with hypoxia produces fluctuations in oxygen levels, leading to ischemic events that induce neovessel proliferation to compensate for low oxygen availability. The OIR model takes advantage of the fact that retinal vessel maturation occurs postnatally, with superficial vessels forming the primary vascularization during the first postnatal week, whereas secondary vascularization is completed around 3 weeks postnatally [12]. Therefore, after birth, retinal vessels display the ability to be modulated by changes in oxygen tension, thus offering the possibility to intervene experimentally in different phases of retinal vascularization. According to the OIR model, exposure to hyperoxia during early retinal vessel maturation results in decreased vascular density, while returning to normoxia, sensed as hypoxia, leads to abnormal neovascularization as a compensatory mechanism in response to low oxygen tension.

Immature retinal vascularization in mouse pups can mimic the retinal vasculature in premature infants, in which the peripheral retina is incompletely vascularized at birth, therefore making it sensitive to changing oxygen levels. In fact, under the normal development of the human fetus, retinal blood vessels, growing from the optic nerve, would reach the peripheral retina at approximately 40 weeks of gestation, while premature birth occurs before 37 completed weeks of pregnancy, with different categories depending on gestational age. Immature retinal vessels can be affected by oxygen tension, with exposure to high oxygen levels contributing to the avascular phase (22–32 weeks of gestation). In particular, a hyperoxic environment sensed after delivery, in comparison with in utero hypoxia, retards the maturation of retinal vessels, which is further delayed by supplemental oxygen. The new capillary vessels suppressed in the peripheral retina create a hypoxic state that generates a pro-angiogenic condition, leading to the vaso-proliferative phase (32–34 weeks of gestation) characterized by abnormal, leaky new vessel overgrowth in the peripheral retina [21].

**Figure 3:**
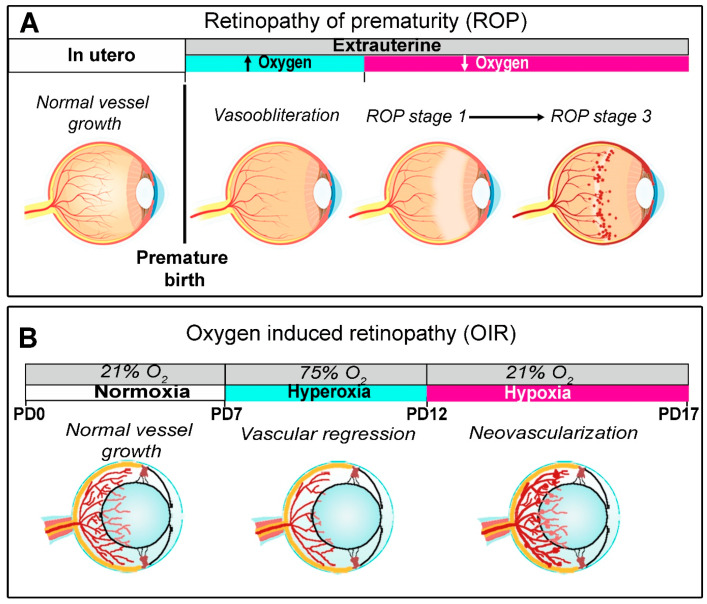
The OIR model of ROP. (**A**) From hyperoxia-induced vaso-obliteration, ROP progresses to stage 1, which is characterized by a net demarcation between a retinal zone rendered avascular by oxygen exposure after premature birth and a still persisting vascular zone. Through stage 2, ROP stage 3 is characterized by progressively overgrowing retinal vessels in response to reduced oxygen tension induced by halted vessel growth. (**B**) In the OIR model mimicking ROP, a normoxic environment from birth to postnatal day (PD) 9 leads to the normal growth of superficial vessels. The following experimental increase in oxygen tension (from PD7 to PD12) leads to vascular regression in the central retina, with the formation of an avascular region. A shift to environmental oxygen tension sensed as hypoxia activates the pro-angiogenic cascade, which causes retinal vessel overgrowth in the midperiphery. Modified from [10].

In Figure 3, major events that characterize ROP stages are reproduced by oxygen manipulation over the different phases of the OIR model.

At the clinical level, ROP is a retinal vasoproliferative disease that affects premature infants, in which low birth weight and hyperoxia exposure after premature birth affect its global prevalence and severity [22]. In the United States, approximately 14,000 preterm infants develop ROP, of which almost 1500 cases progress from moderate to severe stages that require medical care and treatment [23]. In this respect, no significant difference in the trend in ROP prevalence has been found over the last four decades, but its higher prevalence in premature infants ≤ 28 weeks of gestational age is indicative of a strict relationship with improved neonatal care, mostly in low-income populations [22].

Although the OIR model mimics specifically ROP, it can be applied to additional retinal diseases that involve retinal vessel proliferation, including PDR. In diabetes mellitus, for instance, hyperglycemia leads to vessel defects that result in a hypoxic microenvironment with subsequent VEGF accumulation, a major cause of retinal neovascularization [24,25]. Despite the initial stimulus for abnormal vessel growth differing between PDR and ROP, vessel abnormality starts with an initial phase characterized by vascular drop-out and vessel growth cessation, resulting in hypoxic tissue that causes profuse vascular growth misdirected to the vitreous chamber. This complex situation is well reproduced by the OIR model, in which neonatal mice exposed to high levels of oxygen are characterized by the vaso-obliteration of the developing retinal vasculature. Upon return to normoxic conditions, hypoxia-induced factors are upregulated, and neovascularization occurs, leading to the formation of pathological blood vessels [26]. While the OIR model does not involve diabetes, it, however, recapitulates the abnormal angiogenesis that occurs in late DR, making it a valuable tool for studying the mechanisms underlying these processes. The OIR model has been used extensively to investigate potential therapeutics for the treatment of DR, including anti-angiogenic agents, neuroprotective compounds, and anti-inflammatory drugs. By studying the effects of these therapeutics on the retinal vasculature in the OIR model, researchers can gain insights into their potential efficacy for the treatment of DR. Indeed, the increasing incidence of type 2 diabetes is a leading cause of new blindness, with an estimated 37% of patients at risk for PDR [27], which is greatly influenced by good or poor glycemic control and the length of time the diabetes has lasted [28]. Although animal models have played an essential role in advancing diabetes research, mimicking PDR still remains impossible to achieve because of the short life span of rodents. On the other hand, the mouse OIR model, although mimicking neovessel proliferation that characterizes PDR, may not fully recapitulate its complex pathophysiology, particularly in the later stages of the disease. In fact, the mouse OIR model exhibits spontaneous neovessel regression in the late stages, which is consistent with the spontaneous involution of ROP seen in humans [29] but not in human PDR, in which the regression of neovascularization cannot be realized without treatments.

## 4. The Potential Value of Anti-VEGF Therapy

The molecular cascade leading to neovessel proliferation in response to hypoxia is complicated by a broad array of pro-angiogenic and pro-inflammatory factors that are upregulated in response to hypoxia to promote the formation of new vessels in order to satisfy the metabolic requirements of cells deprived of oxygen. In the adult, physiological angiogenesis is a complex multi-step process and involves several signaling pathways coordinated by angiogenesis inhibitors and angiogenic stimulators, which, once dysregulated, lead to pathological angiogenesis that can contribute to the development of various diseases [30]. In the ischemic retina, for instance, pathological angiogenesis results in abnormal vessels growing into the vitreous cavity. They are fragile and leak blood and fluid, resulting in retinal bleeding and scar tissue formation that ultimately makes the retina detach from the back of the eye, potentially progressing to vision loss.

In the molecular cascade leading to neovessel proliferation, a major role is played by specific transcription factors that regulate gene expression, allowing cells to adapt to the low-tension environment. In addition to the stabilization of HIF transcription factors, which are primarily involved in hypoxic responses, other transcription factors, such as the nuclear factor k-light-chain-enhancer of activated B cells (NF-κB) and cAMP response element-binding protein (CREB), are also stabilized under low-oxygen conditions thus playing a role in VEGF upregulation [31,32]. To dissect angiogenesis signaling, much complication is provided by the complex interactions between different transcription factors in regulating gene expression, with HIF-1α regulating the expression of NF-κB, which, on its part, binds to the HIF-1α promoter, thus upregulating HIF-1α mRNA [33]. Although transcription factors are the major contributors to angiogenic processes, other factors are involved in triggering VEGF upregulation in response to low oxygen tension. For instance, transforming growth factor-β (TGF-β), tumor necrosis factor-α (TNF-α), insulin-like growth factor-1 (IGF-1), advanced glycation end products, and oxidative stress are all upregulated during hypoxia to promote the transcription of genes like VEGF, although the underlying mechanisms remain to be clarified. For instance, reactive oxygen species have been demonstrated to induce VEGF transcription through the activation of signal transducers and the activator of transcription factor 3 (STAT3) [34]. Despite the complex interplay of numerous molecular mechanisms underlying new vessel formation, the fact that most of them converge on the upregulation of VEGF is indicative of its major role in neovascularization. Besides promoting endothelial cell proliferation, the released VEGF exhibits significant neurotrophic and protective actions on retinal neurons to the point that its sustained suppression due to anti-VEGF therapy may cause retinal damage, indicating that VEGF signaling is required for retinal cell survival [35]. In particular, Müller glia, the major macroglia in the retina, support the surrounding retinal cells through VEGF production, which, on its part, plays a key role in Müller cell survival through neurotrophin release in diseased conditions [36].

From the past discovery of the pro-angiogenic role of VEGF by Napoleone Ferrara [37] to the role of the HIF transcriptional complex in oxygen sensing and adapting cellular response to low-oxygen conditions discovered by Gregg Semenza [38], a major avenue has been traced for the development of anti-angiogenic therapies. Greater momentum has also been derived from the key role that angiogenesis plays in tumor growth and progression since the seminal discovery of Judah Folkman based on the hypothesis that the inhibition of angiogenic processes could promote tumor remission [39]. If the clinical application of anti-angiogenic therapy has been less fortunate than expected, then the large number of studies on tumor vascularity and angiogenesis inhibitors has revealed the complexity of angiogenic mechanisms, including their dependence on different pro-angiogenic factors [40]. Additional the re-evaluation of VEGF in cancer therapy originates from the fact that tumor cells can utilize the pre-existing vasculature of non-malignant tissues without involving angiogenic mechanisms [41]. In addition, tumor resistance to anti-angiogenic therapies [42] and tumor cell refractoriness to chemotherapy after anti-angiogenic treatment [43] call for additional players in the angiogenic process in cancer. Anti-VEGF therapy, instead, still remains the major therapeutic strategy against proliferative retinopathies, but its limited efficacy and major side effects have led to an urgent need for novel therapeutic approaches. Among pro-angiogenic factors, VEGF contributes only in part to neovessel proliferation, as additional targets in the angiogenic cascade participate in neovascular proliferation. Among them, insulin-like growth factor-1 (IGF-1) contributes to choroidal neovascularization, and its signaling promotes VEGF production [44]. In addition, pro-inflammatory cytokines are key regulators of angiogenesis, acting either directly by increasing endothelial cell proliferation or indirectly by increasing the release of pro-angiogenic factors by inflammatory cells [45]. In this respect, a recent review by Uemura et al. [46] pointed to the involvement of VEGF receptor 1 (VEGFR1) in the inflammatory cascades. Besides its role as a decoy receptor, VEGFR1, once activated by the placental growth factor (PlGF), a member of the VEGF family, induces the production of pro-inflammatory and pro-angiogenic mediators by macrophages and microglia, suggesting the efficacy of anti-PlGF therapy [47].

Among key inflammation-related biomarkers, emerging therapeutic strategies include small-molecule inhibitors targeting novel molecular pathways, which can be used as anti-angiogenic agents instead of the current pharmacological management of retinal angiogenesis. In this respect, topical formulations of tyrosine kinase inhibitors that selectively act on VEGFR1 and VEGFR2 are being developed, with sorafenib efficacy already demonstrated in models of proliferative retinopathies [48]. In addition, recent results from retinal endothelial cells exposed to high glucose support the hypothesis that axitinib, a small molecule inhibiting VEGF receptors, could be a valid candidate for handling diabetic retinopathy through topical administration [49]. Trying to navigate the field of retinal angiogenesis, much confusion is generated by an incredible amount of potential angiogenic targets, spanning from the major regulators of angiogenesis to the most detailed and collateral aspects of the angiogenic cascade. Pro- and anti-angiogenic molecules participate in an intricate system that involves a complex arrangement of different factors, including growth factors, adhesion factors, proteases, extracellular matrix proteins, transcription factors, signaling molecules, etc. In this respect, a separate discussion deserves the efficacy of dietary supplements to counteract retinal angiogenesis, with an ever increasing number of preclinical findings that overcrowd the literature. On the other hand, newly discovered factors with anti-angiogenic activity are reported to display their efficacy partly through VEGF inhibition. Therefore, despite much effort at the preclinical and clinical levels, evidence is still lacking regarding the therapeutic efficacy of targeting additional angiogenic pathways in relation to the conventional VEGF cascade. Anti-VEGF agents still represent the best available treatment for neovascular disorders, even in long-term disease progression, although several issues need to be addressed, including combination therapy to maximize clinical benefits by combining drugs with different modes of action. In addition, more convenient drug delivery systems are needed instead of intravitreal injections. On the other hand, both topical and systemic drug delivery are hampered by the anatomical and physiological barriers that drastically reduce the availability of the administered compounds, despite many efforts to improve the delivery systems and reduce the frequency of intravitreal administrations [50]. An update on targeting angiogenesis can be found in Cao et al. [51].

## 5. Gap Between Preclinical Findings and Therapeutic Development

Since the very beginning, the efficacy of novel treatments, as suggested by preclinical findings, was betrayed by the impossibility of bridging the gap between preclinical results and their clinical application. In this respect, despite promising results from several research groups, the therapy of ocular neovascular diseases still remains confined to intravitreal anti-VEGF treatment, with some cases incorporating anti-inflammatory therapies. However, much concern has been raised by the side effects consequent to repeated intravitreal anti-VEGF injections, such as a constant increase in intraocular pressure leading to endophthalmitis [52]. Even more worrying is the possibility that anti-VEGF therapy may lack efficacy, especially after prolonged administration. Another complication is the compelling importance of the neuroprotective function of VEGF, of which retinal neurons might be deprived after anti-VEGF treatment. In retinal ganglion cells, for instance, the autocrine production of VEGF is crucially involved in their survival, irrespective of the damaging effects of increased intraocular pressure in glaucomatous patients [53]. Preclinical investigations are actively ongoing, mostly with the aim of minimizing the number of intravitreal administrations, either through the development of anti-VEGF therapies with extended release [54] or by combining anti-VEGF therapy with new therapeutic strategies for overcoming anti-VEGF limitations and resistance [55]. In perspective, technological advancements, including port delivery systems, gene therapy, stem cell therapy, and artificial vision, might revolutionize the retinal care sector [56]. Without going too far, best of all would be to develop molecules that, through topical or systemic administration, would normalize upregulated levels of VEGF. Although topical administration is the most common route for the ocular delivery of drugs against diseases of the anterior eye, their low bioavailability due to insufficient corneal permeation and short residence time still prevents them from reaching the market. Major obstacles to drug penetration include the various layers of ocular tissues and the barriers posed by the retinal vasculature. However, in preclinical models, some compounds have shown successful penetration into the posterior segment of the eye after topical delivery, and some have already reached clinical trials in which increased lipophilicity ameliorates compound uptake through the cornea, as in the case of small molecules including tyrosine kinase inhibitors [57]. In addition, improved drug delivery might achieve effective drug concentration in the posterior segment of the eye by topical administration, although there are complications in reaching the posterior segment of the eye to achieve a successful concentration at the target site. If we consider systemic administration, the situation seems to improve slightly, although side effects may be added to its limiting factors. After systemic administration, the entry of drug molecules into the retina and vitreous humor is limited by the blood–retinal barrier made up of RPE and retinal capillary endothelial cells. Despite difficulties in the systemic approach, several new oral medications show preclinical and clinical promise for the management of retinal diseases as standalone treatments or adjuncts to current therapy [58]. In this respect, dietary supplements, although widely reported to efficiently affect neovascular diseases of the eye, need a separate discussion. In fact, translating dietary supplements from rodent models to humans are seriously complicated due to the inability to adjust the effective dosage used in animals to the human situation. Indeed, the short lifespan of the animal model requires dietary supplements at much higher doses to demonstrate their efficacy compared to what happens in human diseases, which take years to develop.

## 6. Our Contribution to Academic Research

The systemic administration of drugs, although easy to manipulate, increases the possibility of side effects due to the larger doses that are needed for drug action, leading, in addition, to a wider spread of the administered compound. Systemic delivery for the treatment of proliferative retinopathies requires the development of small molecules that may reach the posterior part of the eye through blood flow [59]. The drug dose administered systemically should achieve a plasma concentration sufficient for reaching detectable levels in the retina. An additional advantage might depend on the lipophilic nature of the drugs, which may allow them to cross the BRB, thus facilitating their availability. The main preclinical findings from our research group have been indicative of promising systemic therapies that can overcome the limitations of intravitreal anti-VEGF administration.

Since 2007, we have investigated the role of three systems in hypoxia-induced angiogenesis, with the aim of providing additional targets for anti-angiogenic therapies. Of these, the somatostatinergic system and the noradrenergic system act in opposition to regulate VEGF accumulation in response to hypoxia. In the first system, somatostatin or the somatotropin release-inhibiting factor (SRIF) belongs to the class of neuroactive peptides that influence retinal physiology. SRIF exists in two isoforms, SRIF-14 and SRIF-28, consisting of 14 and 28 amino acids, respectively. Of these, SRIF-14, henceforth referred to as SRIF, is synthesized primarily in a subclass of amacrine cells and acts through its receptors (sst_1_-sst_5_) as a neurotransmitter, neuromodulator, or trophic factor [60]. In response to hypoxia, the somatostatinergic system displays reduced activity with decreased levels of SRIF, leading to the lower activation of its major receptor sst_2_, while the noradrenergic system becomes overactivated by ischemic conditions. In the first case, the downregulation of the somatostatinergic system has been demonstrated to participate in the pathological angiogenesis of the retina, while in the beta adrenergic system, increased norepinephrine (NE) release due to the hypoxia-induced overactivation of the sympathetic system acts at the β2 adrenoceptor (β2-AR) to promote retinal vessel proliferation. In this case, the amelioration of the pathological angiogenesis can be obtained by the β2-AR blockade with propranolol [61], while in the somatostatinergic system, sst_2_ activation by its specific agonist octreotide has been found to prevent retinal angiogenesis [62]. Additional investigations were performed in the system formed by the ligand urokinase-type plasminogen activator (uPA) and its receptor uPAR, which plays a major pro-angiogenic function and has emerged as a central player in inflammation. As shown by preclinical studies in rodent models of neovascular eye diseases, uPAR has been found to drastically increase in response to hypoxia, thus indicating the possibility that its inhibition may counteract neovessel proliferation and angiogenesis-associated inflammatory processes [19].

### 6.1. The Somatostatinergic System

**Figure 4:**
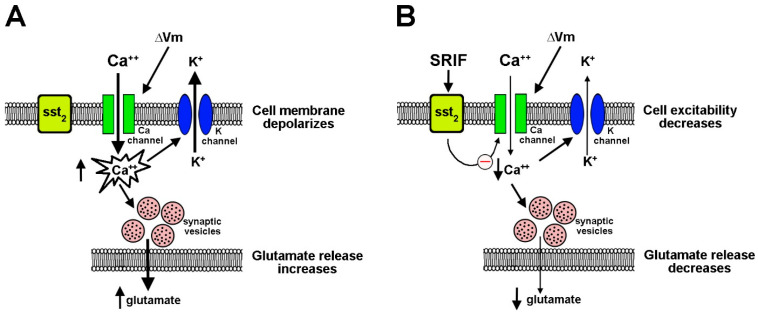
SRIF modulation of glutamate release in rod bipolar cells. (**A**) Glutamate release by rod bipolar cells is mediated by calcium entry through voltage-activated calcium channels, which increases the conductance of calcium-activated potassium channels. (**B**) SRIF binding to sst_2_ inhibits calcium channels, thus reducing calcium entry, which inhibits voltage-dependent potassium channels, ultimately leading to decreased glutamate release. SRIF, Somatotropin release-inhibiting factor; sst_2_, SRIF receptor 2; ΔVm, transmembrane voltage change.

Neuropeptides play a major role in regulating retinal physiology and are also crucial in modulating retinal homeostasis in response to a pathological state. They are mostly localized to wide-field amacrine cells in the inner retina, thus accounting for their major role in regulating retinal stability in response to a changing environment. Among neuropeptides, SRIF is contained in sparsely distributed amacrine cells in the inner nuclear layer (INL) and displaced amacrine cells in the ganglion cell layer (GCL), where it acts at multiple levels of neuronal circuitry by coupling to its distinct receptors [63]. Among them, the sst_2_ receptor is expressed by protein kinase C (PKC)-positive bipolar cells, in which its localization to axon terminals is indicative of the possibility that SRIF may regulate membrane excitability and neurotransmission. Indeed, SRIF has been shown to inhibit calcium influx by influencing voltage-gated calcium channels to protect the retina from excitotoxic damage [64]. The schematic representations in Figure 4 illustrate mechanisms through which SRIF, by activating sst_2_, inhibits excitatory transmission in the retina by exerting modulatory control on glutamate release.

SRIF, in addition to regulating membrane excitability through its coupling to sst_2_ expressed by rod bipolar cells, acts on the sst_2_ expressed by the endothelial cells lining the retinal vessels. In this respect, sst_2_ overexpression in response to hypoxia is likely to represent a compensatory mechanism to protect the retina against ischemic damage, thus implementing the preclinical background for the use of sst_2_ analogs in the treatment of retinal neovascular diseases. In this line, octreotide, an sst_2_ agonist, has been found to prevent hypoxia-induced retinal neovascularization through the inhibition of VEGF accumulation in response to hypoxia [62]. Mechanisms by which octreotide prevents hypoxia-induced VEGF upregulation include the activation of Src homology region 2 domain-containing phosphatase 1 (SHP-1), which dephosphorylates STAT3, a cytoplasmic protein that forms a dimer to translocate into the nucleus, where it activates the transcription of several target genes, including VEGF [34]. In addition to preventing VEGF transactivation, SHP-1 inhibits the synthesis of endothelial VEGF through increased degradation of HIF-1α, thus preventing hypoxia-induced VEGF upregulation [65].

**Figure 5:**
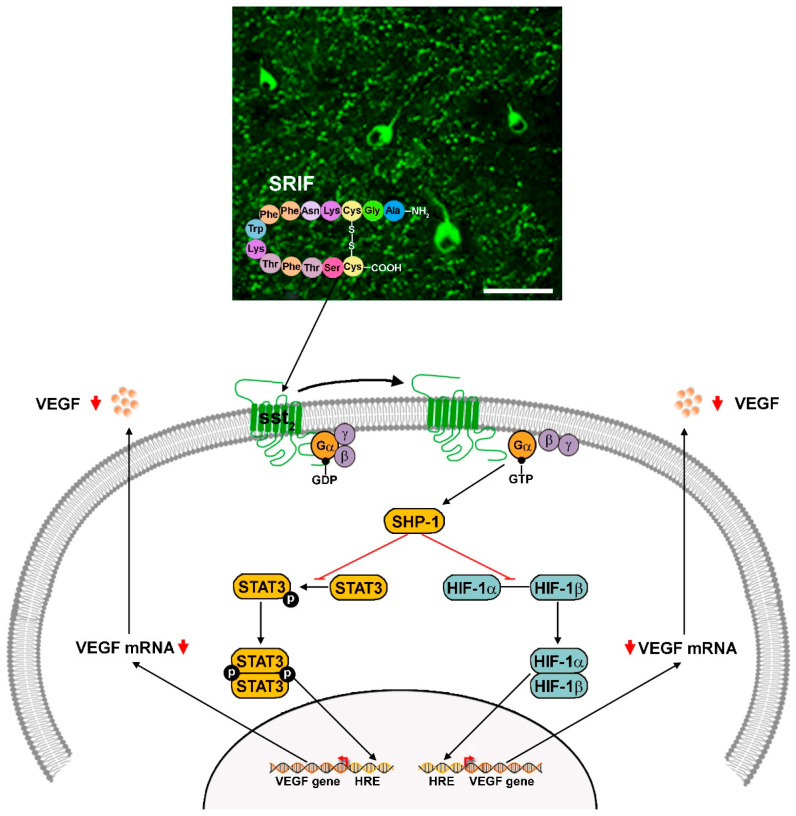
Schematic representation of SRIF inhibitory action on retinal vessel overgrowth in response to hypoxia. The SRIF14 active form, as represented by a cyclic peptide formed from 14 amino acids, is contained in wide-field amacrine cells (immunostained by SRIF antibodies in retinal whole mounts). Scale bar: 100 µm. Modified from [63]. SRIF acts on sst_2_ to activate a molecular cascade, leading to reduced VEGF upregulation in response to hypoxia. sst_2_ interaction with Gα proteins leads to the activation of SHP-1, which inhibits STAT3 phosphorylation and accelerates HIF-1α degradation, finally leading to reduced vessel overgrowth through the downregulated levels of VEGF due to the reduced transactivation of the VEGF gene. SRIF, Somatotropin release-inhibiting factor; VEGF, vascular endothelial growth factor; sst_2_, SRIF receptor 2; GDP, guanosine diphosphate; GTP, guanosine triphosphate; SHP-1, Src homology region 2 domain-containing phosphatase 1; STAT3, signal transducer and activator of transcription 3; HIF-1, hypoxia-inducible factor 1; HRE, hypoxia response element.

In Figure 5, the molecular cascade through which SRIF couples to sst_2_ and inhibits vessel proliferation by reducing VEGF upregulation in response to hypoxia is schematically represented.

SRIF and its analogs have been suggested as a promising therapeutic alternative for the control of proliferative DR and diabetic macular edema (DME) [66,67]. Numerous pre-clinical and clinical trials using SRIF analogs for the treatment of early DR have been initiated. In one such trial (EUROCONDOR), the topical administration of SRIF was found to exert neuroprotective effects but had no impact on the onset of DR, suggesting that SRIF restoration may be especially beneficial for the ever-growing population of patients with early-stage DR [68]. Recently, patients with DME who are resistant to conventional anti-VEGF or anti-inflammatory therapies were found to respond to lanreotide, an octreotide analog acting through a direct anti-VEGF effect or through the inhibition of the IGF-1–growth hormone axis and leading to anti-angiogenic and anti-inflammatory effects [69].

### 6.2. The Beta Adrenergic System

In search of additional factors regulating retinal neovascularization in response to ischemic insult, preclinical studies on the role of the β-AR system in retinal angiogenesis have been ongoing since early 2011. The fact that hypoxia, which is the major cause of angiogenic processes, causes catecholaminergic overstimulation that, in turn, alters signaling pathways associated with β-ARs suggests the possibility that targeting the β-AR system might influence pathological angiogenesis in the diseased retina [70]. We started with the demonstration that, in the OIR model, the blockade of β-ARs with systemic propranolol, a non-specific β1/2-AR blocker, inhibits the upregulated levels of pro-angiogenic factors, retinal vessel proliferation, and blood–retinal barrier breakdown [61], as further confirmed by the topical administration of propranolol [71]. Of the two β-ARs, β2-AR was identified as responsible for the pro-angiogenic effects of sympathetic overstimulation under hypoxia [72], as further demonstrated by the finding that the desensitization of β-ARs consequent to their activation acts by reducing β-AR signaling, as obtained after β-AR blockade [73]. From 2011 to date, in conjunction with preclinical investigations, clinical trials are also ongoing to demonstrate that pharmacologic intervention with propranolol is effective in counteracting the progression of ROP in preterm infants. In particular, exploratory clinical trials using oral propranolol and microdrop formulations showed the efficacy of propranolol in reducing ROP progression and the need for laser photocoagulation or anti-VEGF treatment, despite the risk of some complications due to less developed organs and systems [74].

Despite the efficacy of the β-AR blockade, propranolol was never found to prevent the vaso-obliteration of the central retina in response to hyperoxia. In this respect, β3-AR, the last cloned receptor in the β-AR family, has been recently identified as a new player in the sympathetic regulation of retinal angiogenesis. In fact, it was demonstrated to counteract the pro-angiogenic action of β2-AR by inhibiting pathological angiogenesis in the OIR model through an action on the vaso-obliterative phase [75]. In ROP, the severity of vaso-obliteration in phase 1 largely influences the severity of phases 2 and 3, as a clear indication that preventing vessel loss in the first phase might be of potential therapeutic value [12]. This has been confirmed by the OIR model, in which the disappearance of the capillary network in the central retina after hyperoxia, in combination with astrocyte loss, leads to the formation of an avascular zone that, therefore, determines an ischemic condition causing, in turn, neovessel proliferation in the midperiphery [17]. In this respect, the finding that, in the OIR model, β3-AR agonism prevents hypoxia-driven neovessel proliferation by acting through the recovery of astrocyte loss in the central retina has focused our attention on the link between β3-AR and astrocytes [76]. There are several possibilities that explain the mechanisms underlying the efficacy of β3-AR activation in astrocyte recovery. A likely possibility is that NE released in response to hypoxia activates retinal astrocytes by acting on β3-AR to promote their process formation, which guides the growth of the vascular network. That astrocyte rescue might prevent pathological angiogenesis in the retina has been supported by recent results in the OIR model demonstrating that decreased ferroptosis, a major cause of retinal vaso-obliteration in response to hyperoxia, prevents ischemic damage at the midperiphery through astrocyte recruitment [77]. Acting on the vaso-obliterative phase to prevent vasoproliferation is a particularly attractive strategy, and future studies are ongoing to investigate the mechanisms coupling β3-AR activation to astrocyte recruitment.

**Figure 6:**
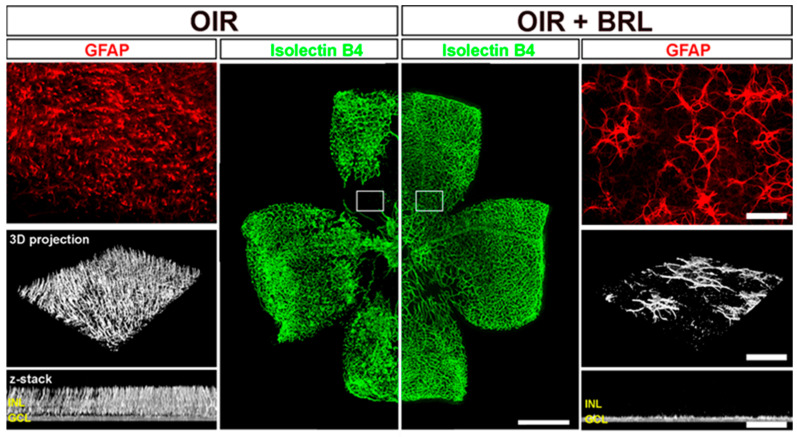
β3-AR involvement in the recovered vascularization of the central retina otherwise made avascular by hyperoxia. Representative images of whole mount retinas stained with isolectin B4 from OIR mice either untreated or treated with BRL (BRL37344). Scale bar: 1 mm. Hyperoxia from PD7 to PD12 leads to vaso-obliteration in the central retina, in which GFAP-IR delineates dense Muller cell terminals that are indicative of Muller cell gliosis, as also determined by the 3D reconstruction and Z-stack projection. In OIR, low oxygen tension consequent to vessel deprivation leads to ischemic conditions, triggering abnormal vessel proliferation in the midperiphery. After the systemic administration of BRL to activate β3-AR, GFAP-IR is found to delineate the profiles of densely recovered astrocytes. Replenished vessels in the central retina help prevent low oxygen tension, thus ultimately counteracting the occurrence of pathological vessel overgrowth in the midperiphery that is instead characterized by healthy superficial vasculature. Boxed areas correspond to high-magnification images of the central retina. Scale bars: 50 μm. GFAP, Glial fibrillary acidic protein; INL, inner nuclear layer; GCL, ganglion cell layer. Modified from [75].

As shown in Figure 6, in the OIR model, the agonism of β3-AR leads to normal vascular regrowth in the central retina, which is otherwise rendered avascular by hyperoxia exposure, with the consequent recovery of the mid-peripheral retina, in which hypoxia-driven neovessel proliferation is prevented. This would occur through astrocyte recovery, although the mechanism underlying β3-AR efficacy in sculpting astrocytes into a template that guides revascularization remains to be clarified.

### 6.3. The UPA-UPAR System

From 2014 to 2020, the potential efficacy of inhibiting the interaction between uPA and its specific receptor uPAR has been actively investigated. In the family of blockers of the uPAR pathway, the peptide Ac-L-Arg-Aib-L-Arg-L-α(Me)Phe-NH2, named UPARANT, acts by preventing the binding between uPAR and formyl peptide receptors (FPRs), which belong to a class of G-protein-coupled receptors with a major role in the regulation of inflammatory/angiogenic responses [78]. At the functional level, UPARANT has been found to prevent neovessel proliferation induced not only by VEGF upregulation, as in the models of retinal angiogenesis [79], but also by the vitreous fluid from patients with PDR, thus interfering with the angiogenic potential of the vitreous expression of cytokines and growth factors [80]. Indeed, inflammation is a significant factor in the pathogenesis of neovascular retinal diseases, and cytokine production by microglial cells reverberates on Müller cells by stimulating their release of additional inflammatory mediators, which contributes to BRB breakdown and vessel leakage [81], suggesting that inflammatory treatments may play a role as an adjunctive therapy in patients who fail to respond to anti-VEGF therapy [82].

**Figure 7:**
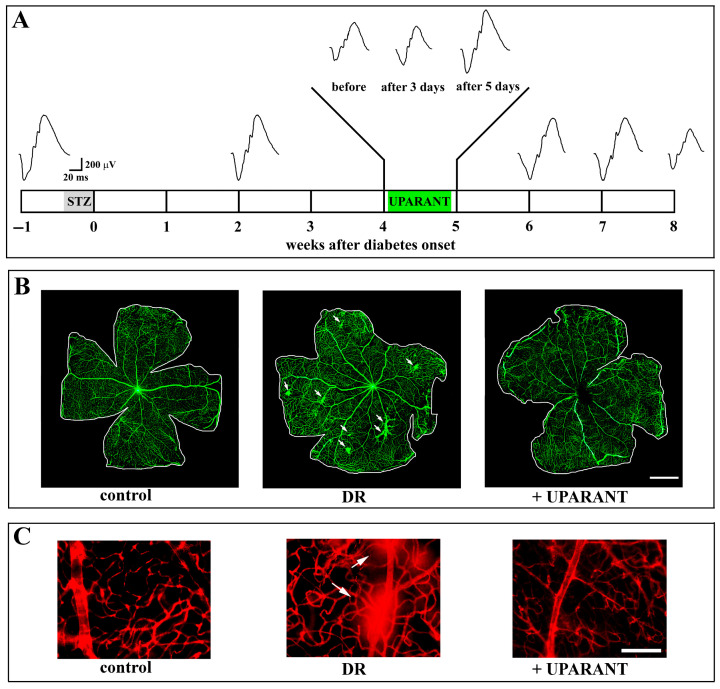
UPARANT recovers dysfunctional electroretinogram (ERG) and blood–retinal barrier (BRB) leakage in models of diabetic retinopathy (DR). (**A**) Longitudinal ERG monitoring is conducted either under normal conditions or at different times after diabetes onset by the injection of streptozotocin (STZ). Four weeks after diabetes onset, the ERG becomes dysfunctional, with decreased amplitudes of the a and b waves. The systemic administration of UPARANT (daily, over 3 days of treatment) does not affect the ERG amplitude, which instead returns to normal after 5 days of treatment. The ERG continues to function for up to 2 weeks, after which it becomes dysfunctional again. In B and C, in conjunction with ERG restoration, UPARANT ameliorates BRB dysfunction, as qualitatively evaluated by fluorescein leakage (**B**) and Evans blue dye (**C**) in control and DR models that are either untreated or treated with UPARANT. White arrows point to extravasation in untreated DR models. Scale bar: 1 mm in B and 200 μm in C. Adapted from [83,84].

After an initial investigation of the effects of UPARANT in the mouse model of OIR, our work progressed with comparable studies in additional models of retinopathies in which UPARANT has been found to prevent microvascular diseases, inner BRB leakage, and visual dysfunction, likely by inhibiting transcription factors regulating pro-angiogenic and pro-inflammatory factors [83]. In particular, the effectiveness of UPARANT as anti-inflammatory therapy is supported by the finding that its systemic administration in models of type 2 diabetes acts by preventing retinal impairment in response to persisting hyperglycemia through reduced levels of uPAR and its membrane partners, thus rendering the treatment more efficient than the acting downstream receptor coupling alone [84]. Figure 7 summarizes the main outcomes of the UPARANT treatment in the models of DR.

In the laser-induced CNV model to mimic neovascular AMD, the systemic administration of UPARANT was effective as its intravitreal injection, suggesting a less invasive administration route [85]. In addition, systemic UPARANT was found to act in a therapeutic regimen by recovering the pathological signs of DR, thus providing evidence of an alternative route to anti-VEGF treatment [83,84]. In this respect, the combined administration of anti-VEGF agents with steroids may offer benefits in treating proliferative retinopathies not only by synergistically acting but also by sustaining the therapeutic effect of the anti-VEGF treatment to overcome the limitation of the repeated intravitreal administration of anti-VEGF drugs. The possibility that the duration of intravitreal anti-VEGF treatment might be extended by the combined systemic administration of UPARANT was supported by preliminary findings in the model of DR. In this model, the intravitreal injection of Aflibercept, a recombinant decoy receptor that sequesters free VEGF [86], has been found to improve both depressed ERG and BRB dysfunction 1 week after its injection, with an efficacy lasting 2 weeks. Aflibercept’s efficacy on both ERG and BRB breakdown was found to persist over time after the repeated administration of systemic UPARANT at a suboptimal regimen, which by itself has no effect, thus implying the possibility of reducing the frequency of anti-VEGF administration. The present results, although preliminary, are indicative of the possibility of using systemic UPARANT as an adjunctive therapy in combination with intravitreal delivery of anti-VEGF drugs. The efficacy of UPARANT to target both pro-angiogenic and inflammatory pathways adds further benefit to the use of combination therapy. Even the maximum inhibition of VEGF, in fact, does not necessarily block neovascularization due to redundant signaling pathways involved in the neovascular process. Thus, the combination of molecules acting on different pathways responsible for pathologic angiogenesis (such as UPARANT) may provide a more profound inhibition of neovascularization than the use of molecules blocking a single pro-angiogenic signal (such as anti-VEGF drugs).

In general terms, the powerful efficacy of combination therapy against neovascular processes in the eye has been demonstrated in both animal models and patients. In DR models, retinal microvascular alterations are greatly improved by combining the intravitreal administration of ranibizumab with molecular tools inhibiting fibrotic processes that contribute to retinal detachment [87]. In addition, the combination of intravitreal bevacizumab with neuroprotective agents reduces abnormal vascular permeability with more efficacy than that of bevacizumab alone [88]. At the clinical level, aflibercept in combination with antibodies interfering with angiogenic pathways different from those activated by VEGF results in a significant increase in the therapeutic efficacy of each monotherapy alone [89]. In addition, treatment with anti-VEGF combined with dexamethasone is a viable alternative to treat both DME and nAMD patients resistant to anti-VEGF monotherapy [90,91]. Finally, combined anti-VEGF administration with steroid therapy is superior to solely anti-VEGF therapy in the treatment of resistant DME [92], with comparable efficacy between treatment-naïve patients and treatment-resistant patients [93].

**Figure 8:**
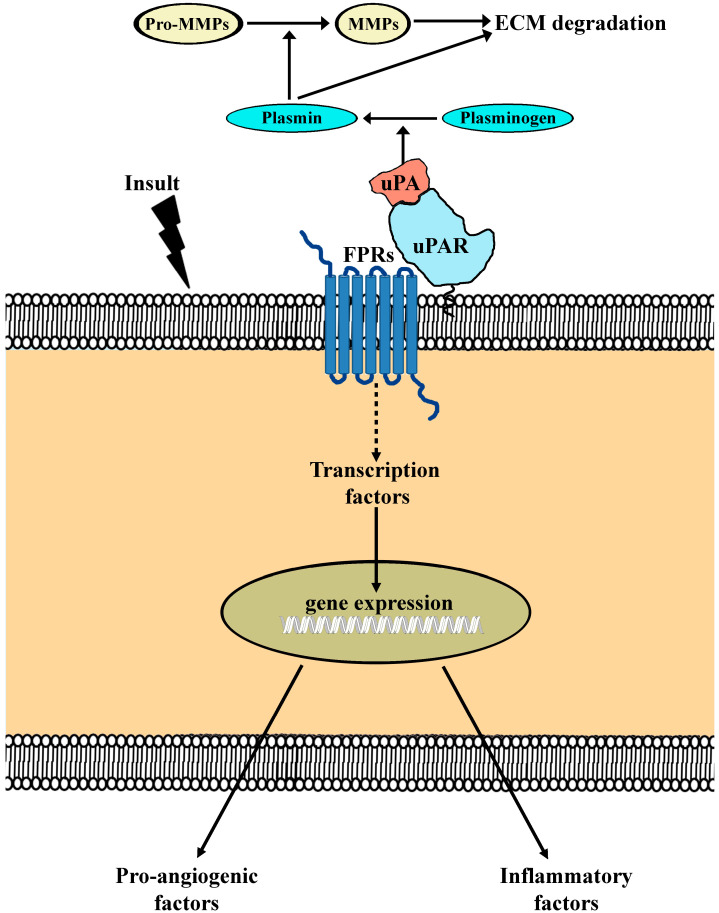
Schematic diagram depicting the mechanisms underlying the effects mediated by the activation of the uPA-uPAR system in response to an ischemic insult. Ischemia-associated upregulation of uPA and uPAR results in increased FPR signaling that causes the overexpression of pro-angiogenic and inflammatory factors through an increased activation of transcription factors regulating their gene expression. Extracellularly, uPA binding to uPAR mediates the process through which plasminogen is transformed into plasmin. Plasmin cleaves and activates MMPs to degrade ECM components. MMPs, Matrix metalloproteinases; ECM, extracellular matrix; uPA, urokinase-type plasminogen activator; uPAR, uPA receptor; FPR, formyl peptide receptor. Modified from [19].

At the molecular level, UPARANT interferes not only with the pro-angiogenic and pro-inflammatory cascade activated by post-receptor signaling, but it also limits stroma invasion by proliferating endothelial cells. uPAR binding to formyl peptide receptors (FPRs) activates multiple signaling pathways mediating the angiogenic and inflammatory response to an ischemic insult. Extracellularly, uPA-uPAR binding induces the production of plasmin that promotes the breakdown of both the extracellular matrix and basement membrane through the activation of proteolytic enzymes, which mediate the degradation of the endothelial cell membrane and extracellular matrix [78]. In Figure 8, signaling pathways mediating uPA/uPAR interaction are schematically represented.

## 7. Technical Methodologies in Proliferative Retinopathies

Over the years, many resources have been dedicated to emerging approaches for closely mimicking human retinal diseases, a critical node for disease intervention. Although animal models are useful tools for elucidating the pathophysiology of proliferative retinopathies, their use is limited by the inability of a single model to mimic the complexity of human diseases, the lack of reproducibility, and the significant differences with human tissues that guide researchers to inquire about other possible options, which has led to in vitro models being explored. In particular, innovative and complex in vitro models that can combine neuronal, glial, and vascular cells in a controlled environment have been developed. They range from traditional monolayer cell cultures to 3D cell cultures to generate tissue or organ-on-a-chip platforms [94]. In the case of DR, for instance, its complexity due to the close interactions among different cell types has led to the development of an in vitro model in which microvascular cell types interact with pericytes and astrocytes to form the inner BRB that regulates the properties of the endothelial cells [95]. A powerful tool for replicating retinal diseases and testing their potential therapies is additionally provided by an in vitro version of the retina derived from advanced stem cell technology that allows the use of pluripotent stem cells to design 3D retinal organoids, which closely mimic the structure and function of the human retina [96]. In particular, an optimized protocol for organoid vascularization allowed for the generation of a complex capillary meshwork that intrudes among retinal layers, thus providing the potential of vascular retinal organoids [97].

In contrast to major advances in disease modeling, methodologies for disease assessment, including the possible benefit of anti-angiogenic treatments, remain confined to conventional techniques, with few exceptions such as the use of optical coherence tomography angiography (OCT-A) to assess the state of the retinal vasculature and the use of ERG in all its facets to assess retinal function in relation to retinal vasculature. After the development of OCT in the human eye [98], the further improvement of OCT techniques allowed the development of OCT-A to visualize retinal blood vessels in patients or animal models [99]. OCT-A is based on the assumption that in the living retina, blood cells are transported by the blood flow; thus, their course through the vessels allows the acquisition of flow maps. Compared to fluorescein angiography or fundus cameras, OCT-A allows for the improved visualization of retinal perfusion. Different parameters in OCT-A images, including vessel density, space between vessels, and perfusion density, are important for monitoring disease progression and the possible benefit of anti-angiogenic treatments. Compared to conventional fluorescein angiography, OCT-A allows for the precise analysis of new blood vessel formation in animal models, although its lateral resolution might be too low to allow the distinction between small and adjacent capillaries.

OCT-A in combination with ERG recording provides a combined view of vascular damage and the neuronal populations affected by the damage. ERG is a non-invasive method used to measure the electrical responses of various retinal cell types to visual stimulation. Each ERG waveform reflects an ensemble response from many neuronal and glial cell populations, and much effort has been dedicated to their role in waveform generation. In this respect, ERG in response to flash stimulation provides information about the activity of the outer retina, whereas the retinal response to a pattern-reversing, black-and-white checkerboard or striped stimulus, or ERG pattern, measures mostly the activity of retinal ganglion cells [100]. Despite electroretinography entering the field of methodologies very early, its application to preclinical studies and clinical practice is still limited, although detecting the functional correlate of vessel impairment might facilitate the determination of structural alterations. In addition, treatment efficacy on vessel recovery does not always reflect ERG recovery, which therefore needs to be assessed.

## 8. Conclusions

Although the OIR model recapitulates major steps in neovessel proliferation with a simplified representation of overgrowing vessels, human neovascular diseases of the retina take years to be diagnosed and cannot be mimicked by animal models. In addition, models of retinal vessel proliferation do not recapitulate the multifactorial etiology of human diseases, which pool together oxidative stress, hypoxia, inflammation, and angiogenesis driven by a combination of genetic and environmental risk factors. As an additional consideration to modeling limitations, treatments that are effective in models do not always translate to clinical efficacy, thus limiting the process of drug development. In this respect, no single animal model may completely cover the complexity of human diseases, thus requiring the need for multiple models to evaluate whether a potential therapy might enter clinical trials, a condition almost impossible to honor at an experimental level. Although new, human-derived in vitro models can recapitulate some aspects of disease processes, animal models still represent a vital step in the translational pathway from the bench to clinical routine.

Since the isolation and cDNA cloning of VEGF in 1989 [37], a main road has opened up for the use of anti-VEGF treatment in neovascular diseases of the retina, although with several limitations. The question is whether more effort should be dedicated to solving those limitations or to discovering novel angiogenic molecules that could replicate the success of VEGF while overcoming its limitations. At the preclinical level, the number of candidates that can support neovessel proliferation in ischemic retinal pathologies is growing day by day, together with new uses for existing drugs and new drug delivery systems that are still receiving increasing attention. For instance, non-degradable implants for the sustained release of anti-VEGF drugs are an advantageous strategy for treating retinal angiogenesis [54]. In addition, combining anti-VEGF monotherapy with additional systemic therapies to combat angiogenesis and inflammation may overcome anti-VEGF resistance and improve anti-VEGF efficacy [101].

Inhibiting retinal vessel proliferation may not be enough to save visual function, as retinal cell degeneration is indeed a major endpoint of blinding diseases. Yet, despite exciting advances in neovessel recovery, there continues to be a critical need for the development of neuroprotective strategies to promote retinal cell survival in conjunction with repaired neovascularization. In this respect, a major role is played by the tight interplay between vessels and retinal cells through glial elements [102]. Although glial activation and dysfunction have been shown to participate in the neovascular disease of the retina, their contribution needs further elucidation in order to be ultimately translated into novel clinical therapies. If disrupted interactions among neurons, vascular cells, and glia, which collectively form the neurovascular unit, are associated with retinal disease progression, then novel treatments targeting the components of the neurovascular unit should ameliorate retinal structure and function [103]. In particular, targeting glial cells by regulating the secretion of cytokines from activated microglia or by modulating glial cell association with retinal vessels might prevent persistent inflammation that exacerbates retinal angiogenesis [45]. Despite the increasing interest in exploiting glial targets, no drug specifically targeting glial cells is on the market yet. In close association with activated microglia, astrocytes play a major role in regulating retinal vascularization. Despite the pivotal role of astrocytes, their targeting remains unavailable so far, mostly with respect to the possibility of inhibiting astrocyte over-activation, which could be a new target for the treatment of retinal neovascular diseases [104]. In this respect, reducing microglial activation by anti-inflammatory treatments might inhibit the transition of astrocytes into their pro-inflammatory phenotypes [45].

In view of the potential of treating the components of the neurovascular unit, a better understanding of its impairments and the establishment of therapies for neurovascular protection should be helpful in finding new ways of managing proliferative retinopathies. For instance, reversing a dysfunctional neurovascular unit might protect retinal cells from degeneration. On the other hand, the fact that any neuroprotective treatment may efficiently act on early neuronal defects, for which their detection instead occurs in advanced stages of diseases, has limited the development of neuroprotective care. The inability to detect retinal diseases at their earliest signs is likely to prevent patients from benefiting from neuroprotective therapies that could delay or prevent late-occurring vision loss. In this respect, a major problem is the lack of screening methods that are sensitive enough to detect neovascular diseases at their early stages, especially when they are correlated with systemic pathologies or are simply age-dependent [105]. The early detection of proliferative diseases of the retina might ensure timely intervention before substantial structural damage and sight-threatening complications occur. In this context, a potential use of artificial intelligence might optimize the automated detection of small retinal alterations before their progression, thus allowing for the best options for preventive treatments [106]. In addition, in the case of systemic diseases such as diabetes, the identification of ocular and systemic biomarkers is crucial to ensure timely intervention before the occurrence of structural and functional complications [107].

Despite future advancements in the field of neovascular diseases of the retina, at present, it is difficult to weigh the pros and cons of preclinical findings and their potential translation into human applications. The fact that patients are still reliant on a therapy that dates back to the early 2000s is disappointing when one considers the extent of the commitment at the preclinical and clinical levels.

## Data Availability

No new data were created or analyzed in this study. Data sharing is not applicable to this article.
